# Clinical significance of miR-155 expression in breast cancer and effects of miR-155 ASO on cell viability and apoptosis

**DOI:** 10.3892/or.2012.1634

**Published:** 2012-01-12

**Authors:** SHU-RONG ZHENG, GUI-LONG GUO, WEI ZHANG, GUAN-LI HUANG, XIAO-QU HU, JIN ZHU, QI-DI HUANG, JIE YOU, XIAO-HUA ZHANG

**Affiliations:** 1 Department of Oncology, The First Affiliated Hospital of Wenzhou Medical College, Zhejiang 325000; 2 Department of Breast Surgery, Jiaxing Maternity and Child Health Care Hospital, Zhejiang 314000, P.R. China

**Keywords:** breast neoplasms, microRNAs, microRNA-155, clinicopathological features, antisense oligonucleotide, viability, apoptosis

## Abstract

Accumulating evidence shows that mircroRNAs (miRNAs) play a vital role in tumorigenesis. miR-155 is one of the most multifunctional miRNAs whose overexpression has been found to be associated with different types of cancer including breast cancer. To further determine the potential involvement of miR-155 in breast cancer, we evaluated the expression levels of miR-155 by real-time PCR and correlated the results with clinicopathological features. Matched non-tumor and tumor tissues of 42 infiltrating ductal carcinomas and 3 infiltrating lobular carcinomas were analyzed for miR-155 expression by real-time PCR. Further, we used an antisense technique to inhibit miR-155 expression *in vitro*. WST-8 test was performed to evaluate cell viability and apoptosis assay was used to investigate the effect of the miR-155 antisense oligonucleotide (miR-155 ASO) on HS578T cell death. The expression levels of miR-155 were significantly higher in tumor tissues than the levels in matched non-tumor tissues (P<0.001). Up-regulated miR-155 expression was associated with lymph node positivity (P=0.034), higher proliferation index (Ki-67 >10%) (P=0.019) and advanced breast cancer TNM clinical stage (P=0.002). Interestingly, we next found that miR-155 expression levels had close relations with ER status (P=0.041) and PR status (P=0.029). Transfection efficiency detected by flow cytometry was higher than 70%, the WST-8 test showed that viability of HS578T cells was greatly reduced after transfection with miR-155 ASO compared with the scramble (SCR) group or the liposome group. The Annexin V-FITC/PI assay also indicated that transfection with miR-155 ASO promoted apoptosis.

## Introduction

miRNAs are small [20–24 nucleotides (nt)] non-coding RNA gene products that have become known as important regulators of various cellular processes by post-transcriptionally modulating gene expression (1,2). Currently, more than several hundred unique mature human miRNAs are known (3). miRNAs have been reported to be involved in tumorigenesis acting as oncogenes (4–6) or suppressors (7,8). Several other reports have described altered expression of miRNAs in cancer tissues compared to normal tissues, suggesting that these miRNAs could potentially represent novel clinical and prognostic markers (9–12). In particular, miR-155, which is considerd as an oncomiR, has been found to be up-regulated in B cell lymphoma and carcinoma of head and neck, breast, lung, pancreas, kidney and colon (9,13–17).

To date, several studies have demonstrated the association of elevated miR-155 with late stage and poor overall survival in several types of malignancy (9,18). It is well known that the prognosis of breast cancer patients is closely related with the clinicopathological features including tumor size, histological grade and the presence of lymph node or distant metastases (19,20). These clinicopathological features alone or in combination, enable the identification of individuals who are at increased risk of dying of breast cancer and also who may benefit from aggressive treatment (21). However, there is rare evidence indicating the correlation of miR-155 expression with clinicopathological features in human breast carcinoma, which may help us understand the role of miR-155 in this disease.

Therefore, in this study, we used the real-time RT-PCR method to detect the expression of miR-155, which we correlated with clinicopathological features in 42 infiltrating ductal carcinomas and 3 infiltrating lobular carcinomas. Additionally, in order to analyze the effect of miR-155 on cell growth and to investigate the potential mechanism of miR-155 in breast cancer, we utilized the antisense technique to inhibit miR-155 expression *in vitro*.

## Materials and methods

### Cell line and breast tumor specimens

The breast cancer cell line (HS578T) was obtained from ATCC and grown according to ATCC recommended culture conditions. Fresh samples from 45 cases of human breast cancer and paired normal adjacent tissues (>5 cm from cancer tissue) were obtained from the Department of Oncology, The First Affiliated Hospital of Wenzhou Medical College (Wenzhou, China) between February 2009 and June 2009. The samples used were not subjected to preoperative radiotherapy and/or chemotherapy and all patients were treated by modified radical mastectomy. Before RNA extraction, sections were stained with H&E for histological diagnosis and tumor cell evaluation. Only those cases with a population of at least 70% tumor cells in the section were used in this study. Tissues were preserved by snap-freeze and stored at −80°C. The diagnosis and histological grade of each case were independently confirmed by two pathologists based on the WHO classification. The clinical stage was classified according to the American Joint Committee on Cancer (AJCC) tumor-lymph node-metastasis (TNM) classification system (22). The relationship between the clinicopathological characteristics of the patients and miR-155 expression are summarized in Table II.

### Synthesis of miR-155 ASO sequences and transfection

The mature miRNA sequences are available from the miRNA registry. The sequences of miRNA ASO were designed, according to the principle of sequences complementary to the mature mRNA. The ASO and the scrambled negative control (SCR) sequences used in this study are listed in Table I. Both of them were chemically synthesized and 2′-OMe modified by Shanghai GenePharma Co., Ltd. (Shanghai, China) and stored at −20°C. Twenty-four hours before transfection, HS578T cells in the exponential phase of growth were seeded in 96- or 6-well plates (Costar) and allowed to grow overnight. The cells were then transfected with oligonucleotides using Lipofectamine™ 2000 reagent (Invitrogen) in Opti-MEM I for 6 h. Transfection complexes were prepared according to the manufacturer’s instructions. At the end of transfection, the cells were incubated in medium containing 10% fetal calf serum (FCS). Transfection efficiency was detected by flow cytometry.

### Total RNA extraction and real-time PCR for quantitative analysis of miR-155

HS578T cells were incubated in 6-well plates and transfected with 75 nM oligonucleotides using the Lipofectamine 2000 reagent for 24 h. The same process was followed as described above. Total RNA from the tissues or treated HS578T cells was isolated using TRIzol reagent (Invitrogen) according to the manufacturer’s instructions. RNA purity and concentration were controlled by UV spectrophotometry (A260:A280>1.8).

The primers for the analysis of miR-155 expression were designed by the Shanghai Sangon Biological Engineering Technology and Services Co., Ltd. (Shanghai, China) and are summarized in Table I. Mixtures of 1 μg of total RNAs together with 50 nM reverse primer, 2 units of RNAase inhibitor (Takara Bio), 5 units of M-MLV reverse transcriptase (Takara Bio) and 0.5 μM dNTP were used for each RT reaction. The reaction parameters were incubation at 25°C for 10 min, 42°C for 60 min, 52°C for 15 min, 70°C for 15 min and then a hold at −20°C. At the same time, to generate the cDNA template for the endogenous control PCR reactions, first strand cDNA was synthesized using 1 μg of RNA from the same samples for stem-loop reverse transcription and oligo(dT) as the primer. The reaction parameters were incubation at 42°C for 30 min, 70°C for 15 min and then a hold at −20°C.

The qPCR was performed on the Applied Biosystems 7500 detection system. For quantitation of miR-155, the 25 μl PCR included 1 μl of the RT product of miR-155, 1X SYBR-Green I Mastermix (Toyobo Co., Ltd.), 0.5 μM specific forward primer of miR-155 and 0.5 μM reverse primer. For the endogenous control, U6 snRNAs, 1 μl of cDNAs synthesized by using oligo(dT) were used as a template. The reaction parameters were incubation at 95°C for 10 min, then 40 cycles of 95°C for 15 sec and 60°C for 1 min. To calculate the relative concentration, miR-155 and U6 CT values for all samples were obtained. A normalized expression for each sample was obtained by dividing the Ct value of miR-155 by the same sample’s U6 Ct and designated as ΔCt. This value was then transformed by the formula 2^−ΔCt^. Furthermore, the (ΔΔCt) method was used in comparing miR-155 expression in each group of treated HS578T cells or matched non-tumor tissue to cancer tissues.

### Cell viability and apoptosis assays

The effect of miR-155 ASO on HS578T cell viability was determined by the 2-(2-methoxy-4-nitrophenyl)-3-(4-nitrophenyl)-5-(2,4-disulfo-phenyl)-2H-tetrazolium, monosodium salt (WST-8) assay kit (CCK-8, Dojindo, Kumamoto, Japan). Twenty-four hours before transfection, 1×10^4^ HS578T cells/well were seeded in 96-well plates and allowed to grow overnight. The cells were then transfected with three different concentrations of miR-155 ASO (25, 50 and 75 nM) and the highest concentration of SCR siRNA (75 nM) using Lipofectamine 2000 according to the manufacturer’s protocol. After 24 h, WST-8 was added into each well for 1 h before the measurement according to the manufacturer’s instructions. The absorbance at 450 nm was measured by a microplate reader. The following formula was used for calculating the inhibition rate. Inhibition rate = (1-absorbance of treated cells/control cells) × 100% (23). The apoptosis assay was performed with the Annexin V-FITC/PI Apoptosis Detection kit (Roche). HS578T cells were transfected with miR-155 ASO or SCR siRNA as previously described for 6 h, and incubated in medium containing 10% FCS for another 24 h in 6-well plates. Cells were collected and double-stained with FITC-conjugated Annexin V and propidium iodide (PI). For each sample, data from approximately 1×10^4^ cells were recorded in the list mode on logarithmic scales. Apoptosis and necrosis were analyzed by quadrant statistics on PI-negative, Annexin V-positive cells and both positive cells, respectively.

### Statistical analysis

For all data, statistical analysis was performed in SPSS 17.0 for Windows (SPSS, Inc.). The miR-155 expression levels were characterized by their median and ranges from the 25th to the 75th percentile. The Wilcoxon test was used for comparing two paired groups (tumor and paired non-tumor), the Mann-Whitney U test for two independent groups and the Kruskall-Wallis test for three independent groups (relationship between miR-155 expression level and TNM stage of breast cancer). The one-way ANOVA test was performed to investigate the differences in the obtained results of the WST-8 array and apoptosis analysis. All tests were two-tailed and the significance level was set at P<0.05.

## Results

### Expression of miR-155 in tumor tissue and matched non-tumor tissue

**Figure 1:**
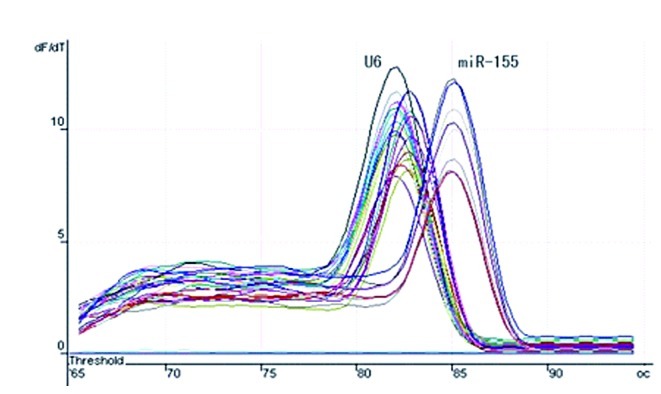

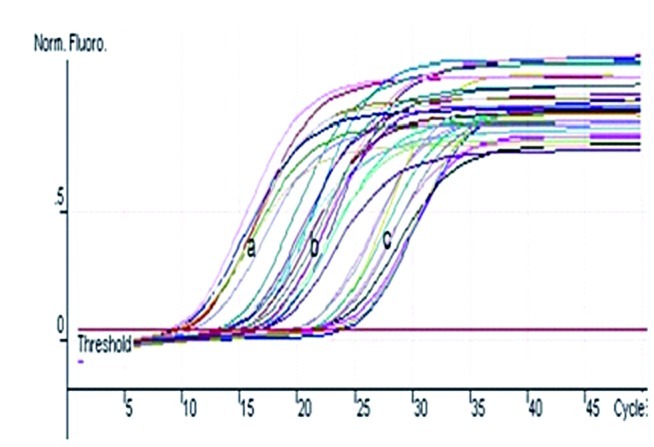

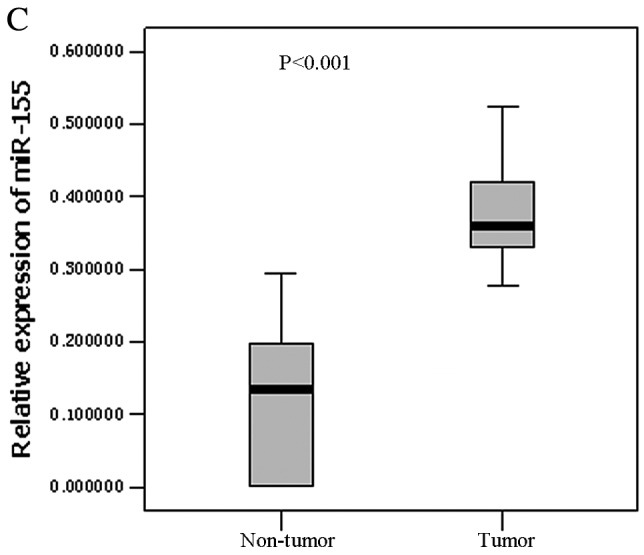
miR-155 expression in breast cancer. (A) The melting-curves of miR-155 and U6 snRNA are presented as a single, sharply-defined melting curve with a narrow peak, indicating that pure, homogeneous PCR products were produced. (B) Representative amplification curves show that the Ct values of miR-155 in the tumor tissue are lower than in the non-tumor tissue, indicating the miR-155 expression is higher in tumor tissue than in non-tumor tissue. Reactions were performed in triplicate. (A) U6 snRNA of tumor and matched non-tumor; (B) miR-155 of tumor; (C) miR-155 of matched non-tumor. (C) Differences in the expression levels of miR-155 between the tumor tissue and matched non-tumor tissue (box-plot diagrams with median, 1st quartile, 3rd quartile and non-outlier range).

The melting-curves of miR-155 and U6 snRNA were sharply defined curves with a narrow peak (Fig. 1A), indicating that pure, homogeneous PCR products were produced. As shown in the amplification curves (Fig. 1B), the Ct value of miR-155 in tumor tissue was lower than that of miR-155 in non-tumor tissue, which means the expression level of miR-155 in tumor samples was higher than that in the controls. The median of the relative expression of miR-155 (2^−ΔΔCt^) was 0.360 (25th-75th percentile, 0.328–0.420) in tumor samples, with that in non-tumor control samples set at 0.135 (25th–75th percentile, 0.003–0.199) (Fig. 1C). The difference of expression of miR-155 between the tumor and the control samples was statistically significant (P<0.001, Wilcoxon test).

### Association between miR-155 expression level and clinicopathological parameters

The up-regulated expression of miR-155 was associated with advanced clinical TNM stage (P=0.002, Kruskall-Wallis test), lymph node positivity (P=0.034, Mann-Whitney U test) and high proliferation index (Ki-67 >10%) (P=0.019, Mann-Whitney U test). Furthermore, the miR-155 expression levels were closely related to ER and PR status (P=0.041 and 0.029, respectively, by the Mann-Whitney U test). However, no significant relationship was found between the expression of miR-155 and the menstrual status (P=0.640), size of primary tumor (P=0.530), Her-2 status (P=0.647) and pathologic type (P=0.559) using the Mann-Whitney U test (Table II).

### miR-155 ASO down-regulation of miRNA expression

**Figure 2:**
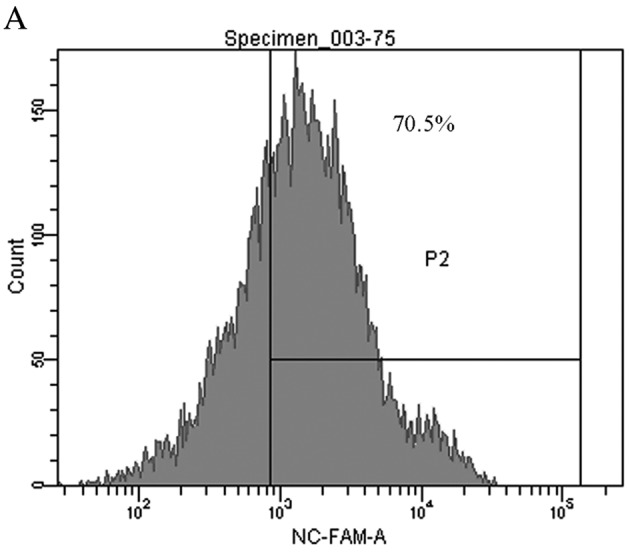

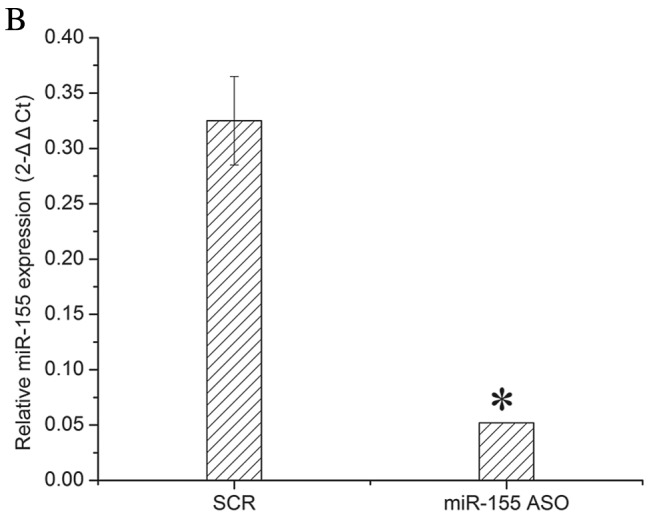
Detection of transfection efficiency and miR-155 expression ratio relative to U6 snRNA in HS578T cells. (A) Eight hours after transfection with 75 nM 5′FAM SCR, the transfection efficiency was detected by flow cytometry. The P2 region stands for the number of cells that were successfully transfected with 5′FAM SCR by Lipofectamine 2000. (B) HS578T cells were transfected with 75 nM miR-155 ASO or SCR. Total RNA from treated cells was extracted by TRIzol and quantified by ultraviolet spectrophotometry. miR-155 and U6 snRNA expression were determined by quantitative real-time PCR, according to the manufacturer’s instructions. Results showed that miR-155 ASO down-regulated miR-155 levels in HS578T cells. ^*^P<0.01, compared with the SCR group.

Eight hours after transfection with 75 nM 5′FAM SCR, the transfection efficiency was detected by flow cytometry (Fig. 2A).To validate whether miR-155 ASO decreased miR-155 levels in treated HS578T cells, miR-155 and U6 snRNA expression was determined by real-time RT-PCR. The fold change for the miR-155 expression level was calculated using the 2^−ΔΔCt^ method. As shown in Fig. 2B, the 2^−ΔΔCt^ value of HS578T cells treated with anti-miR-155 was 0.052, which showed that the level of miR-155 in HS578T cells was down-regulated by miR-155 ASO.

### miR-155 ASO inhibition of HS578T cells viability

**Figure 3:**
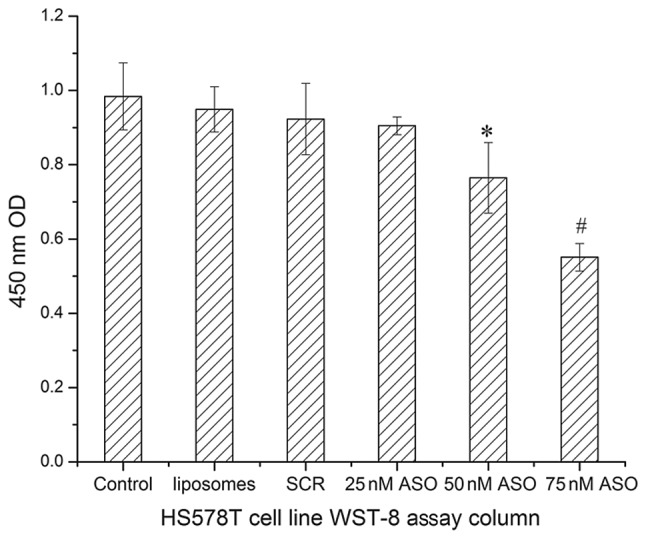
WST-8 assay on control, liposomes, SCR and test groups.^*^P=0.000 compared to control group; ^#^P=0.000 compared to control group.

In this study, we determined the influence of miR-155 ASO on cell viability by WST-8 assay. Optical densities at 450 nm were obtained for 4 groups: 0.984±0.090 for control group, 0.949±0.061 for liposomes group, 0.923±0.096 for SCR group and 0.905±0.024, 0.765±0.095 and 0.551±0.037, respectively for 25, 50 and 75 nM concentrations of miR-155 ASO (Fig. 3). These results demonstrated that the optical density and therefore the cell viability was similar in the control, SCR, 25 nM miR-155 ASO and liposome groups (P=0.237, one-way ANOVA test). However, there were significant differences between the optical density in the 50 nM miR-155 ASO and 75 nM miR-155 ASO group and the control group (P=0.000 for both by one-way ANOVA). The optical density and cell viability gradually decreased with the increase of miR-155 ASO concentration (0.905±0.024, 0.765±0.095 and 0.551±0.037, respectively). At 75 nM concentration of miR-155 ASO, the optical density and cell viability were nearly half of these parameters in the control group. These data indicate that a higher concentration of miR-155 ASO had a higher toxicity effect on HS578T cells and could decrease the cell viability and proliferation.

### miR-155 ASO promotion of HS578T cell apoptosis

**Figure 4:**
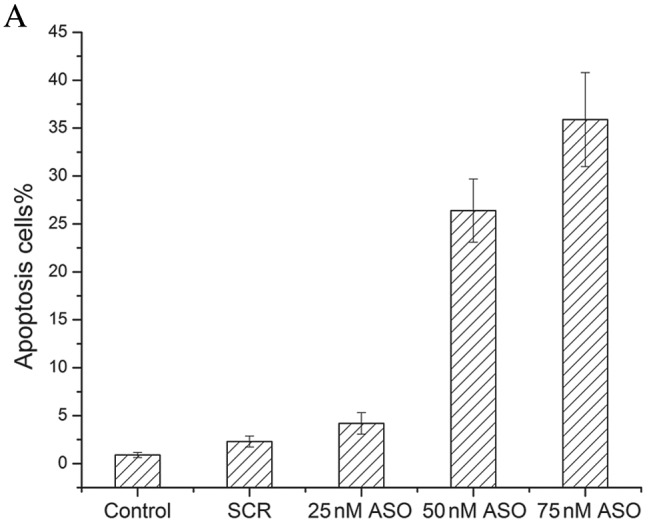

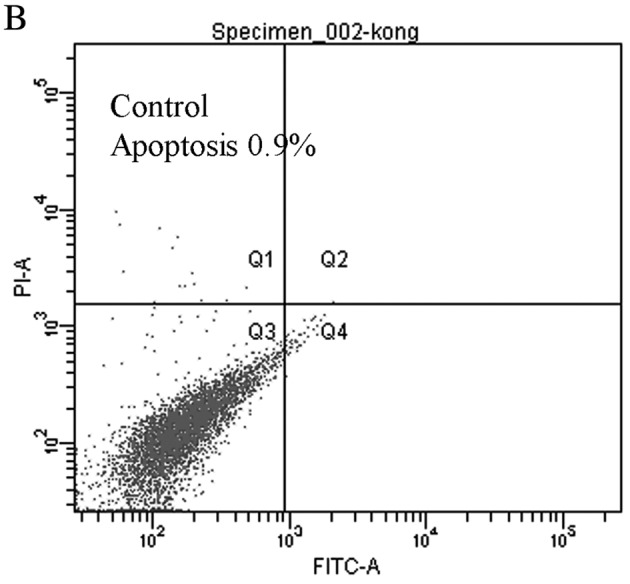

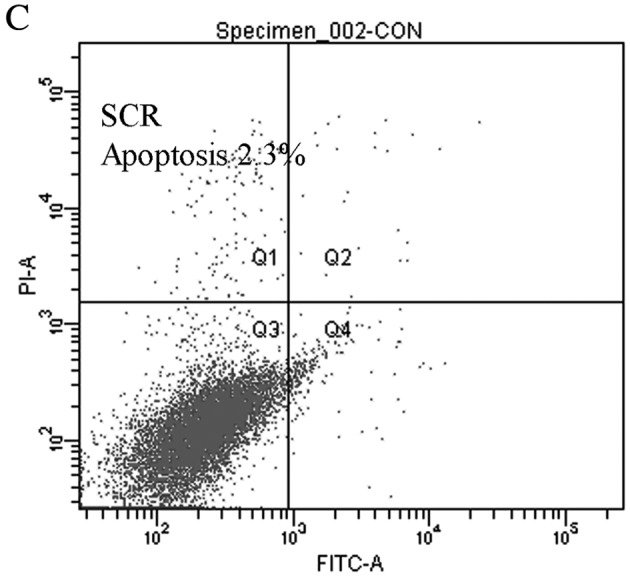

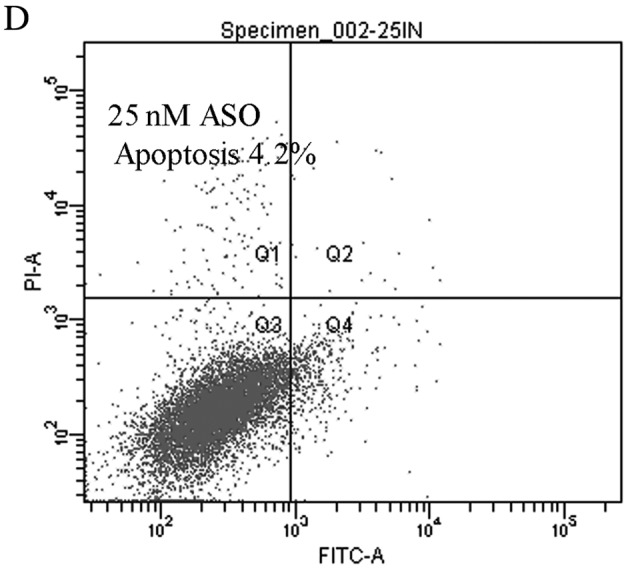

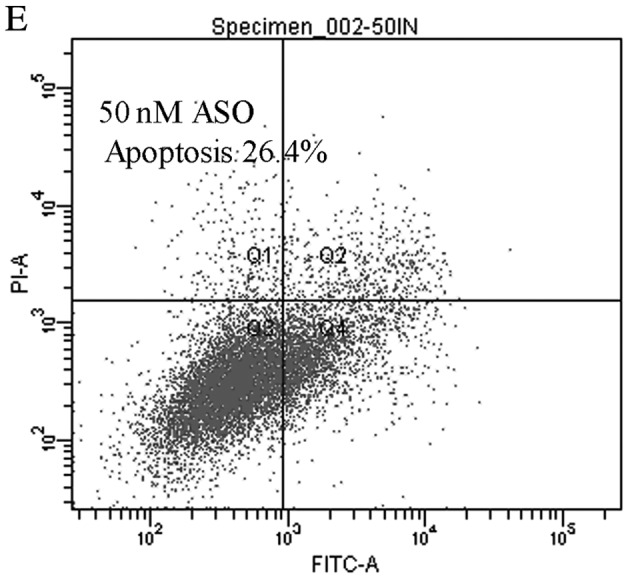

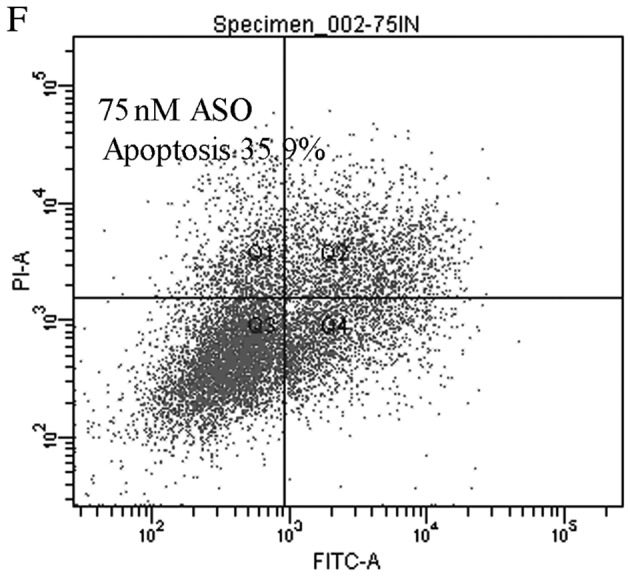
Induction of HS578T cell apoptosis by miR-155 ASO. (A) HS578T cells were transfected with miR-155 ASO or SCR for 6 h and incubated in medium containing 10% FCS for another 24 h. Apoptosis of HS578T cells were double-stained with Annexin V and PI, and detected by flow cytometry. miR-155 ASO 50 and 75 nM could efficiently induced apoptosis (P<0.01 as compared with SCR). (B-F) The images of apoptosis were analyzed by double staining with Annexin V and PI.

To explore the effects of miR-155 ASO on cell apoptosis, miR-155 ASO treatment was investigated in HS578T cells. Apoptotic HS578T cells were detected by double staining with Annexin V and PI. The results demonstrated that miR-155 ASO could induce cell apoptosis. Along with the increase of concentration of miR-155 ASO, the apoptosis rate of HS578T cells gradually increased. The double-stained images are shown in Fig. 4.

## Discussion

As previous research has indicated, miR-155 acts as a multifunctional miRNA in many pathophysiological process, such as immunology (24,28), inflammation (25,26), hematopoiesis (27), angiocardiopathy (28) and carcinogenesis. Interestingly, miR-155 may act as a bridge between inflammation and malignancy, which may provide new insight in carcinogenesis (29–31). miR-155 is one of the most prominent miRNAs involved in tumor development and progression. Diverse studies have shown that miR-155 is overexpressed in various tumor types. Not surprisingly, it was found to be significantly up-regulated in breast cancer in both our and previous other studies (15,29,32). However, there is rare evidence focused on the relationship between miR-155 expression and clinicopathological features in breast cancer.

In the first stage of our study, we detected the expression of miR-155 in all 45 carcinomas and paired non-tumor tissues by real-time RT-PCR. The amplification and melting curve, as shown in Fig. 1, indicate the method is specific and sensitive enough for detection of miR-155. Furthermore, to reduce the error caused by gene expression differences between different individuals, we used matched non-tumor tissue as control and used 2^−ΔΔCt^ to represent the level of miR-155 expression in tumors relative to matched non-tumor samples. As expected, our results confirmed that miR-155 expression was significantly up-regulated in breast cancer compared with the matched non-tumor tissue.

Our study next focused on the potential correlation between the expression level of miR-155 and various breast cancer clinicopathological characteristics. The data showed that high levels of miR-155 appear to be significantly correlated with advanced clinical stage, lymph node metastases, and higher proliferation index (Ki-67 >10%). All of them are well known as poor prognostic factors of breast cancer patients (33–35). Interestingly, we next found that patients with ER-positive or PR-positive tumors have higher miR-155 expression levels than those that are ER-negative or PR-negative. This is in part consistent with the findings of Zhu *et al* (36) and Lu and Tsourkas (37), whose experimental material was either serum or cells but not tissues. However, it should be noted that both ER and PR are protective factors of patients with breast cancer (38–40). Therefore, the representation of the oncomiR-like miR-155 is contradictory to the protective function of ER and PR. And we may hypothesize that it is miR-155 that results in the aggressive behavior in carcinomas that ER- or PR-positive. These results indicate that, as an independent risk factor, miR-155 could serve as a prognostic marker for survival of breast cancer patients.

The precise molecular mechanisms behind the altered expression of miR-155 in breast cancer remain poorly understood. To our knowledge, this is the first report to describe the significance of miR-155 to the clinical stage, lymph node metastasis, hormone receptor status of breast cancer patients. It was recently reported that miR-155 mediates lymphoblastoid cell lines (LCLs); suppression of this miRNA, which is highly expressed in LCLs, was associated with decreased cell proliferation and increased apoptosis (41). In solid tumors, such as breast cancer and lung cancer, similar findings of the function for miR-155 were obtained (29,42). Furthermore, Kong *et al* (15) discovered that miR-155 is a critical therapeutic target and is closely related with chemosensitivity in breast cancer. Two recent studies reported two additional direct miR-155 targets, FOXO3a (15) and suppressor of cytokine signaling 1 (29), respectively, both of which function as protective factors in breast cancer patients, demonstrating that this miRNA acts as an oncomiR in breast cancer.

Therefore, in this study, we utilized miR-155 ASO for repression of HS578T cell growth and proliferation. Transfection of HS578T cells with either miR-155 ASO or SCR were performed successfully with at least 70% efficiency. The results of real-time PCR suggested that the synthesized miR-155 ASO could effectively down-regulate this miRNA expression. The WST-8 assay was performed and the results indicated that although 25 and 50 nM of miR-155 ASO have a toxic effect on HS578T cells, 75 nM of miR-155 ASO could strongly repress tumor cell proliferation. Moreover, apoptosis analysis demonstrated that the apoptosis rates of the group of 50 and 75 nM of miR-155 ASO were significantly higher than the other three groups.

In conclusion, we demonstrated that overexpression of miR-155, one of the most significantly altered miRNAs in breast cancer, is related to clinical stage, lymph node metastasis, higher Ki-67 and hormone receptor status of breast cancer patients. Although the precise molecular mechanism of the ectopic expression of miR-155 in breast cancer requires further clarification, our data suggest that miR-155 may be a promising candidate as a molecular biomarker and a potential therapeutic target for breast cancer intervention.

## Figures and Tables

**Table I:** Sequences of oligonucleotide and primers for the analysis of miR-155 expression.

Gene name	Sequence (5′→3′)
miR-155 ASO	ACCCCUAUCAAGAUUAGCAUUAA
5′FAM SCR	CAGUACUUUUGUGUAGUACAA
Stem-loop RT primer	CTCAACTGGTGTCGTGGGGCAATTCAGTTGAGCCCCTATC
miR-155-F	TGCCTCCAACTGACTCCTAC
miR-155-R	GCGAGCACAGAATAATACGAC
U6 snRNA-F	CTCGCTTCGGCAGCACA
U6 snRNA-R	AACGCTTCACGAATTTGCGT

F, forward primer; R, reverse primer.

**Table II:** Relationship between the miR-155 expression level and clinicopathological parameters of breast cancer.

Variable	N	Relative expression of miR-155^a^	P-value^b^
Menstrual status			0.640
Premenopausal	20	0.365 (0.333–0.421)	
Post-menopausal	25	0.355 (0.308–0.403)	
Tumor size (cm)^c^			0.530
≤2	19	0.356 (0.316–0.401)	
>2	26	0.361 (0.334–0.420)	
TNM stage			0.002
I	8	0.316 (0.286–0.350)	
II	24	0.358 (0.332–0.416)	
III	13	0.417 (0.367–0.446)	
ER status			0.041
Positive	30	0.367 (0.349–0.424)	
Negative	15	0.318 (0.299–0.401)	
PR status			0.029
Positive	26	0.398 (0.354–0.423)	
Negative	19	0.335 (0.313–0.399)	
Her-2 status			0.647
Positive	14	0.398 (0.313–0.420)	
Negative	31	0.359 (0.337–0.419)	
Lymph node status			0.034
Metastasis	20	0.383 (0.355–0.437)	
No metastasis	25	0.355 (0.314–0.399)	
Proliferation index (Ki-67) (%)			0.019
≤10	21	0.353 (0.364–0.398)	
>10	24	0.387 (0.355–0.437)	
Pathologic type			0.559
Ductal	42	0.360 (0.331–0.420)	
Lobular	3	0.330 (0.277–0.442)	

a Median of relative expression, with 25th–75th percentile in parentheses.

b P<0.05 was considered significant (Mann-Whitney U test between 2 groups and Kruskall-Wallis test for 3 groups).

c Maximal tumor diameter.
